# A causal link between mental imagery and affect-laden perception of climate change related risks

**DOI:** 10.1038/s41598-023-37195-w

**Published:** 2023-06-21

**Authors:** Hulda Karlsson, Erkin Asutay, Daniel Västfjäll

**Affiliations:** 1 Department of Behavioural Sciences and Learning, JEDI-Lab, Linköping University, House I:3, Campus Valla, 581 83 Linköping, Sweden; 2 Decision Research Eugene, Oregon, USA

**Keywords:** Psychology, Human behaviour

## Abstract

Previous studies have shed light on the importance of affect in risk perception and the role of mental imagery in generating affect. In the current study, we explore the causal relationship between mental imagery, affect, and risk perception by systematically varying the level of mental imagery in three levels (i.e., enhanced, spontaneous, or prevented). In light of the increasing environmental risk of adverse events caused by climate change, we operationalize risk as participants' perceived risk of climate change. One-thousand-fifty-five participants were recruited online and randomized to one of three levels of mental imagery. As predicted, we found a causal link between the level of mental imagery, affective experience, and perceived risk of climate change, in that enhanced mental imagery caused a larger decrease in positive affective valence and a larger increase in perceived risk of climate change. We argue that mental imagery enhances the negative affect associated with the risk event by creating a perceptual experience that mimics seeing the environmental risk events.

## Introduction

Every day we face different risks, everything from driving in traffic to societal issues like climate change. But what determines how we act in the face of these risks? Why do we sometimes take a leap of fate (i.e., risk seeking), and other times take the safe route (i.e., risk aversive)? The last couple of decades has shed light on the enormous importance of affect in how we perceive risk^1^. The affect-as-information hypothesis stipulates that affective experiences are used as information in judgments and decisions^2^, and the risk-as-feelings hypothesis and the affect heuristic stipulates that affective experiences impact how we perceive risk^1,3^. In this study, we examine a critical, but largely untested, mechanism of the affect heuristic—that there is a causal relationship between mental imagery, affect, and risk perception. To do this we experimentally vary the level of mental imagery associated with the risk of climate change (e.g., floods increasing) in three levels; spontaneous, enhanced and prevented. We suggest that mental imagery (either by increasing the availability of and/or attention to affective cues) can amplify affective experiences and which, in turn, are used as information when making subsequent risk assessments^4,5^.

Climate change is an ongoing crisis that poses several risks for individuals and society. Risky environmental events are already at a high level and are only predicted to increase over the coming decades^6^ and subsequently, these events are predicted to increase the number of climate-change-related deaths to 250,000 deaths per year (between 2030 and 2050)^7^. Thus, it is essential to understand how people perceive potential environmental risk events. Previous research has found that affect plays an essential role in how we perceive the risk of climate change, concerning for example air pollution from the burning of wood for heating^8,9^, and floods^4^.

Throughout the affect-as-information and risk-as-feelings literature, imagery (both pictorial and mental) has often been stipulated to be important for activating risk-associated affects^3,8,10–18^. Mental imagery can be described as a weak form of perception^19^, that enables us to construct a reality. In the same way, that our personal history can influence us, mental imagery can shape our assessments and actions^20^. One potential pathway for mental imagery to impact our assessments and actions is through affect. Indeed, mental imagery, relative to verbal processing^21^, has been shown to have a larger influence on emotions, and it has been proposed to act as an amplifier for emotion and motivation^22,23^.

Although mental imagery has been suggested to impact our affects and subsequent risk perception, few studies have attempted to show a causal relationship between imagery, affect, and risk perception^10,15^. Previous research found that imagining negative consequences of risks (i.e., ignoring persistent medical problems) increased the negative affect, which in turn increased perceived risk^15^. Further, it has also been shown that mentally imagining smoking a water pipe tobacco impacted both the valence (i.e., positive/negative) and risk (i.e., perceived harm of water pipe tobacco use)^10^. Thus, previous research has found support for a causal link between mental imagery, affect, and risk perception in other contexts than climate change. In addition, a link between mental imagery (i.e., episodic future thinking), climate change risk perception, and pro-environmental behaviors (i.e., energy saving, beach cleanup, vegetarian food) has been identified^15^, however in this case leaving out the affective experience in the causal model. Further, affective imagery has been found to have predictive value regarding climate change related risk perception, however only in a cross-sectional design^14^.

Beyond extending the understanding of the causal relationship between mental imagery, affective experience, and risk to a new context (i.e., climate change), our methodology differs from previous research concerning both experimental manipulation and control conditions. The current study manipulates mental imagery into three levels (1) by not specifying how to process the presented environmental events (i.e., spontaneous imagery), or (2) prompting participants to create vivid mental images of the events (i.e., enhanced mental imagery), or (3) preventing vivid mental images through a visuospatial interference task (i.e., prevented mental imagery).

In our enhanced mental imagery condition, we aim for a methodology that creates vivid multimodal mental images. Previous studies have used free or semantic associations^13,24^, as well as imagining or listing consequences of risky behaviors^15,17^. However, approaches for creating vivid mental imagery like the one used in the current research have also been applied^24^. Concerning the control condition, previous attempts have contrasted negative imagery with having no alternative task^10^, completing an arithmetic problem^15^, imagining positive consequences^15^, or cognitively processing the risk (i.e., writing about it)^24^. Having no alternative task or asking participants to cognitively process the risk cannot experimentally represent the absence of mental imagery, since most people spontaneously engage in mental imagery (it is very seldom that people do not, i.e., Aphantasia^25^). Further, contrasting negative and positive mental imagery can only experimentally control for the valence of the imagery, but cannot discern the role of mental imagery itself. Lastly, contrasting mental imagery with an arithmetic task will decrease the focus on presented risks, but might still leave room for visuospatial mental imagery.

In the current study, we instead use a prevented mental imagery condition, utilizing visuospatial interference, which benefits from the limited resources of working memory^26^ and provides visuospatial information that competes with the visuospatial information from mental imagery^19,27,28^. We compare the effect of mental imagery level on affective valence, arousal, and perceived risk of climate change.

### Research questions and hypotheses

Specially, we aim to answer if mental imagery impacts the perceived risk of climate change; and, in addition if mental imagery is a step in the causal relationship between affective experiences and climate change related risk perception. We expected that the magnitude of the change in the dependent variables (perceived risk, affective valence and arousal) from pre-manipulation to post-manipulation too be dependent on condition. Specifically, we expected that:

1. Enhancing mental imagery would lead to a within-subject increase in perceived risk concerning climate change, from pre-manipulation to post-manipulation, that were of larger magnitude compared to both the spontaneous and prevented use of mental imagery (i.e., significant interaction effects).
2. Preventing mental imagery would lead to a within-subject decrease in the magnitude of change in perceived risk concerning climate change, from pre-manipulation to post-manipulation, compared to both the spontaneous and enhanced use of mental imagery (i.e., significant interaction effects).
3. Enhancing mental imagery would lead to a within-subject decrease in the level of positive affective valence and a within-subject increase in the level of arousal, from pre-manipulation to post-manipulation, that were of larger magnitudes compared to both the spontaneous and prevented use of mental imagery (i.e., significant interaction effects).
4. Preventing mental imagery would lead to different magnitudes of change in the within-subject level of positive affective valence and arousal from pre-manipulation to post-manipulation, compared to both the spontaneous and enhanced use of mental imagery (i.e., significant interaction effects [All hypotheses were somewhat altered from the pre-registration to clarify that we expected to find interaction effects]).

## Method

### Participants

Informed consent was collected from all participants. According to guidelines from the Swedish research council concerning the Ethical Review of Research Involving Humans (SFS 2003:460), approval from an ethics committee is not required for behavioral research such as this study.

Data from a total of 1055 participants were collected. Due to exclusions (not completing the full experiment (*N* = 54), long completion time (*N* = 1 [The completion time (14,608.00 s) of the participant was a clear outlier from the mean (*M* = 809.20, *SD* = 672.86)]), the sample used in the analyses consisted of 1000 participants (95% of the recruited sample).

The participants (39.8% female, 58.6% male, 1% non-binary/third gender, 0.6% prefer not to say; no difference between conditions, *p* = 0.53; mean age = 38.65, *SD* = 13.21; no difference between conditions, *p* = 0.81) were recruited online (Prolific) from an American sample and responded through an online survey tool (Qualtrics). All data were collected during a single session (20th of October 2022). To be included in the study participants had to be at least 18 years of age and have an approval rate of 95% on the platform. Participants received monetary compensation for their participation (£ 1.95).

An a priori power analysis was conducted using G*Power, the expected sample size (*N* = 1005) entering the study would allow the detection of small (*f* > 0.10) group differences (1 − β = 0.80) with an alpha value of 0.0125 (corrected for multiple comparisons).

### Procedure and materials

#### Pre-manipulation measures

After giving consent and completing demographic variables, all participants rated the three dependent variables, perceived risk of climate change, and their affective experience in the form of valence, and arousal.

The main dependent variable is the perceived risk of climate change posed to oneself (two items), to others (two items), and to non-human populations on earth (two items), with six items in total on a visual analogues scale (VAS scale) ranging from 0 (Climate change does not present any risk) to 100 (Climate change presents an extreme risk).

Valence was measured using a single item assessing the participants' level of pleasantness. Arousal was also measured with a single item assessing their level of activation. Participants first read a text explaining both the valence scale and arousal scale and then answered the question, “How do you feel right now?” For valence, participants answered on a VAS scale ranging from 0 (Negative) to 100 (Positive), for arousal they answered on a VAS scale ranging from 0 (Low activation) to 100 (High activation).

After the pre-manipulation ratings, the participants were randomized to one of three conditions: (1) spontaneous mental imagery (control), (2) enhanced mental imagery, and (3) prevented mental imagery (the time spent on the experiment varied between conditions, *F* (2, 997) = 44.89, *p* < 0.001). The spontaneous mental imagery condition took the least amount of time (10.31 min), followed by the prevented condition (13.42 min), and the enhanced condition (16.50 min).

Participants in the spontaneous mental imagery condition (control) first read “On the coming pages you will be presented with environmental events that could happen. Please read each environmental event carefully and then move to the next page and answer the questions.” Following this they were presented with an environmental risk event (i.e., water pollution increasing), but received no further instruction on how to process it and were then asked to rate their, valence, arousal, and vividness of their mental images of the environmental risk event. The vividness of mental imagery measure read “How clearly did you see the *environmental risk event* [i.e., water pollution increasing] in your mind’s eye” and participants responded on a five-point scale ranging from 1 (No image at all [only “knowing” that you are thinking of it]) to 5 (Perfectly clear and as vivid as normal vision; adapted from Vividness of Visual Imagery Questionnaire [VVIQ]^29^). The procedure was repeated for a total of four environmental risks events (i.e., water pollution increasing, polar ice caps melting, wildfires increasing, and floods increasing [The order of when the environmental risk events were presented varied according to a Latin Square. No main effects or interactions were found for the order, see supplementary 2]). The environmental risk events were chosen since these have been shown to lead to an active focus on the event^5^.

The spontaneous mental imagery condition (control) was designed to simulate turning people’s attention towards an environmental risk in day-to-day life. Even though we did not specify how to process the environmental risk events, we expect that the manipulation would activate some mental imagery processes. Although the prevalence and intensity of spontaneous mental imagery varies in the population^30^, there are very few people who do not spontaneously use imagery (i.e., Aphantasia^25^). Thus, this condition represents the normative reaction to environmental risk event cues.

#### Enhanced mental imagery task

Participants in the enhanced mental imagery condition were presented with an environmental risk event and were then instructed to create a mental image of that risk, “[example water pollution increasing] Now, please build up a very vivid image of a lake, the sea, or a river, using all your senses. Please imagine before your mind’s eye that this place is filled with trash and debris…” (Material adapted^31–34^). After completing the mental imagery task participants rated experienced valence and, arousal and, vividness of mental imagery. The procedure was completed for all four environmental risk events.

#### Visuospatial working memory interference task

Participants in the prevented mental imagery condition were presented with an environmental risk event and then partook in a mental rotation task, that served the purpose of visuospatial working memory interference. The mental rotation task consisted of completing five mental rotation items. Participants received a tutorial before starting the mental rotation task. They also rated their valence, arousal, and vividness of mental imagery after the mental rotation task for each environmental risk. The procedure was completed for all four environmental risk events.

#### Post-manipulation ratings

Following this, all participants rated the dependent variables again and completed some exploratory measures; pro-environmental donation behavior; pro-environmental intention; pro-environmental impact-belief; anticipated warm glow; severity of environmental risks presented in the study; personal knowledge of the place imagined (for the complete survey, see Open Science Framework). Participants pro-environmental intention was measured with eight items, for example, “How likely is it that you will hang all your clothes on a clothesline, the next time you wash your clothes?”, and answered on a visual analogue scale- (VAS) scale ranging from 0 (Much more likely that I would do the non-environmental behavior (e.g., put them in the tumble dryer) to 100 (Much more likely that I would do the pro-environmental behavior). Their pro-environmental impact beliefs were also measured for the same eight behaviors as in the intention measure, participants answered “How environmentally effective do you think it would be to dry all your clothes on a clothesline rather than putting them in a dryer?” The participants rated all items on a VAS scale ranging from 0 (Will not make any difference at all) to 100 (Will make an extremely big difference).

Participants' responses to the manipulation check and their vividness ratings both indicate that the extent of mental imagery used, and the vividness of mental images created, are in line with what was expected from our manipulations (see supplementary 1 and 3). Although there is a risk of demand characteristics (i.e., cues for the experimental hypothesis), we used a between-subject design where any demand characteristics were constant over conditions, why a demand effect should not have interacted with the effect of the manipulations.

## Results

**Figure 1:**
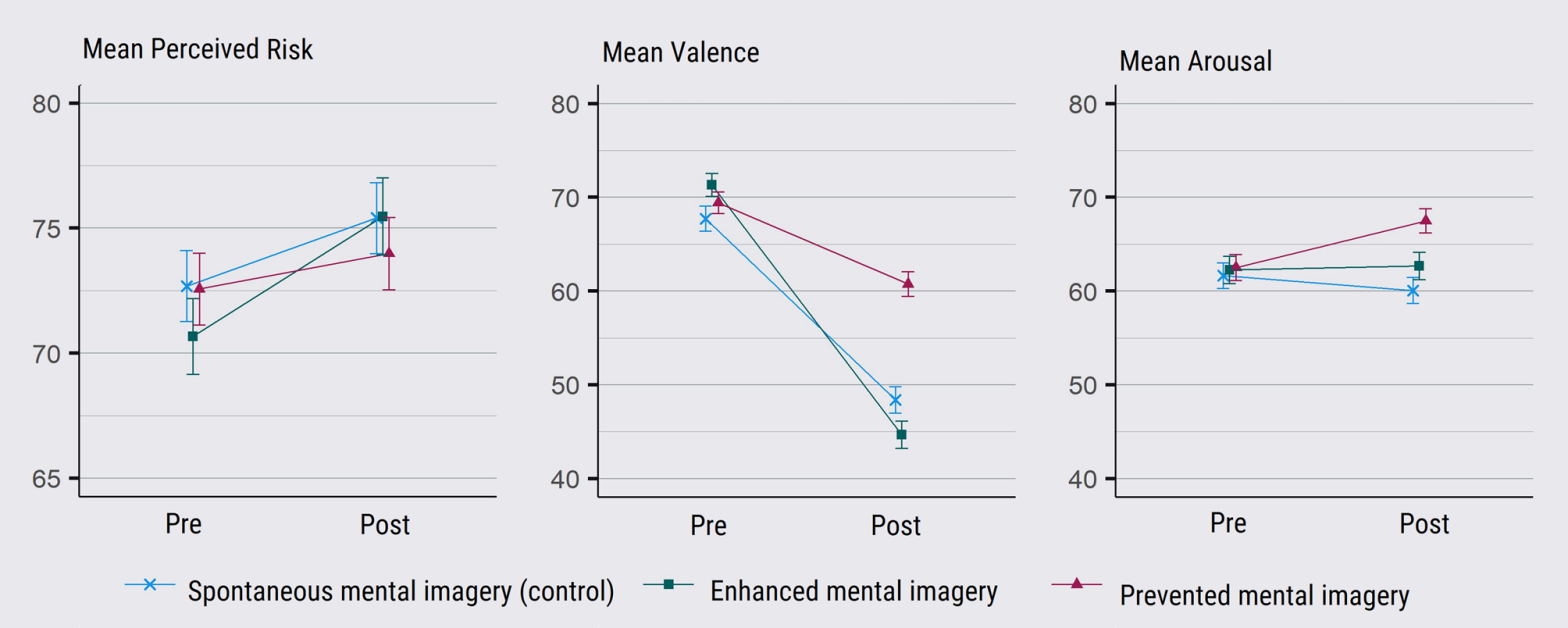
Mean values for perceived risk, affective valence, and affective arousal, presented for pre- and post-manipulation, split per condition. Mean perceived risk presented on different scales (see y-axis) from the mean valence and mean arousal. The values presented for perceived risk are based on the average value at each time. Error bars represent the standard error. This figure includes all participants that were used in the main analysis (*N* = 1000).

For the main analyses on perceived risk, an aggregated measure of perceived risk was used (*α* = 0.82 [Cronbach's alpha for perceived risk was calculated on the change scores (time 2 – time 1)]). No pre-manipulation differences were identified in any of the dependent variables, risk perception (*p* = 0.56), valence (*p* = 0.128), and arousal (*p* = 0.90). All dependent measures deviated significantly from a normal distribution. Since we were mainly interested in the interaction effects, the pre-registered parametric analyses were conducted. Nonparametric tests showed a similar pattern of results for between and within analyses, see supplementary 4). See Table 1 and Fig. 1 for means per variable and condition. We corrected for multiple comparisons (Bonferroni-Holm); all significant values presented remained significant after correction.

**Table 1:** Mean values for perceived risk, affective valence, and affective arousal, presented for pre- and post-manipulation, split per condition.

Measure	Spontaneous mental imagery (control) (*N* = 346)			Enhanced mental imagery (*N* = 308)			Prevented mental imagery (*N* = 346)		
	Time 1	Time 2	Effect size	Time 1	Time 2	Effect size	Time 1	Time 2	Effect size
Perceived risk	72.68 (25.43)	75.41*** (26.34)	0.41	70.67 (26.62)	75.47*** (27.15)	0.53	72.57 (26.69)	73.99*** (26.91)	0.28
*CI (95%)*	69.89, 75.49	72.61, 78.16		67.65, 73.63	72.40, 78.49		69.70, 75.73	71.07, 76.77	
Affective experience									
Valence	67.73 (24.85)	48.40*** (26.22)	0.86	71.35 (21.55)	44.71*** (25.63)	1.03	69.43 (21.60)	60.76*** (24.22)	0.46
* CI (95%)*	65.10, 70.32	45.73, 51.15		68.89, 73.71	41.85, 47.58		67.14, 71.70	58,25, 63.28	
Arousal	61.65 (25.68)	60.07 (25.73)	0.08	68.28 (25.50)	62.68, (25.50)	0.02	62.51 (25.88)	67.50*** (24.05)	0.29
* CI (95%)*	58.96, 64.30	57.38, 62.78		59.47, 65.10	59.83, 65.49		59.66, 65.31	64.93, 70.00	

The risk perception values presented are based on the average value at each time. The values in brackets are the standard deviation. The 95% confidence intervals (CI) are calculated using bootstrapping (10 000 samples). Table 1 includes all participants that were used in the main analysis (*N* = 1000). ***p value < 0.001 indicating significant change between times 1 and 2 for each condition and variable using a repeated measure ANOVA. Effect sizes reflect the change in each condition between times 1 to 2, measured in Cohens.

### Perceived risk of climate change

We expected that enhancing mental imagery would lead to an increase in the perceived risk of climate change, that were of larger magnitude compared to both the spontaneous and prevented use of mental imagery (1). Subsequently, we also expected that preventing mental imagery would decrease the magnitude of change in perceived risk concerning climate change, compared to both the spontaneous and enhanced use of mental imagery (2). To test these hypotheses, we used split-plot ANOVAs 3 (condition) × 2 (time) to examine between- and within-subject differences, with planned comparisons. To answer our hypotheses our main interest was the interaction effects between condition and time, to see if the magnitude of the change in the variables is different depending on the condition (see Table 2 for main analyses for all three dependent variables).

**Table 2:** Split-plot ANOVAs 3 (condition) × 2 (time) with planned comparisons.

Variable	Comparison	Effect	F	df	p	ƞ^2^_p_
Perceived risk	All	Main effect time	177.20	1, 997	< 0.001	0.151
		Main effect condition	0.13	2, 997	0.881	< 0.001
		Interaction effect	18.89	2, 997	< 0.001	0.037
	Control vs enhanced	Main effect time	146.60	1, 652	< 0.001	0.184
		Main effect condition	0.23	1, 652	0.636	< 0.001
		Interaction effect	11.21	1, 652	< 0.001	0.017
	Control vs prevented	Main effect time	84.23	1, 690	< 0.001	0.109
		Main effect condition	0.15	1, 690	0.702	< 0.001
		Interaction effect	8.38	1, 690	0.004	0.012
	Enhanced vs prevented	Main effect time	119.63	1, 652	< 0.001	0.155
		Main effect condition	0.01	1,652	0.920	< 0.001
		Interaction effect	35.47	1, 652	< 0.001	0.052
Valence	All	Main effect time	661.06	1, 997	< 0.001	0.399
		Main effect condition	12.33	2, 997	< 0.001	0.024
		Interaction effect	53.72	2, 997	< 0.001	0.097
	Control vs enhanced	Main effect time	593.49	1, 652	< 0.001	0.477
		Main effect condition	< 0.01	1, 652	0.982	< 0.001
		Interaction effect	15.00	1, 652	< 0.001	0.022
	Control vs prevented	Main effect time	319.21	1, 690	< 0.001	0.316
		Main effect condition	17.68	1, 690	< 0.001	0.025
		Interaction effect	46.32	1, 690	< 0.001	0.063
	Enhanced vs prevented	Main effect time	406.16	1, 652	< 0.001	0.384
		Main effect condition	19.49	1, 652	< 0.001	0.029
		Interaction effect	105.27	1, 652	< 0.001	0.139
Arousal	All	Main effect time	3.87	1, 997	0.049	0.004
		Main effect condition	2.79	2, 997	0.062	0.006
		Interaction effect	9.42	2, 997	< 0.001	0.019
	Control vs enhanced	Main effect time	0.48	1, 652	0.491	0.001
		Main effect condition	0.80	1, 652	0.372	0.001
		Interaction effect	1.34	1, 652	0.248	0.002
	Control vs prevented	Main effect time	5.56	1, 690	0.019	0.008
		Main effect condition	5.39	1, 690	0.021	0.008
		Interaction effect	20.63	1, 690	< 0.001	0.029
	Enhanced vs prevented	Main effect time	11.53	1, 652	< 0.001	0.017
		Main effect condition	1.95	1, 652	0.164	0.003
		Interaction effect	8.37	1, 652	0.004	0.013

The risk perception values presented are based on the average value at each time. Table 2 includes all participants that were used in the main analysis (*N* = 1000).

Comparing perceived risk in all three conditions revealed a significant main effect of time, participants perceived the risk to be more severe post-manipulation than pre-manipulation, no main effect of condition was identified. However, the expected interaction effect between condition and time was significant, indicating that the change in perceived risk varied in strength between the conditions. When comparing the conditions level for perceived risk post-manipulation with a simple main effect analysis (Sidak), no difference was found between either the control and enhanced condition (*p* = 1.00), control and prevented condition (*p* = 0.86), or the enhanced and prevented (*p* = 0.86).

To explore the interaction effect further, we conducted our planned comparisons, first between the spontaneous mental imagery condition (control) to the enhanced condition. We again found a significant main effect of time, no main effect of condition, and a significant interaction effect. Although both the control and enhanced condition increased perceived risk of climate change after exposure to environmental risk event cues, the enhanced mental imagery increased perceived risk more than the control condition as hypothesized.

Subsequently, we conducted our second planned comparison, between the spontaneous mental imagery condition (control) and the prevented condition. We again found a significant main effect of time, no main effect of condition, and an interaction effect. Again, both the control and prevented condition increased perceived risk of climate change, but as hypothesized the prevented mental imagery condition increased the perceived risk less than the control condition.

Lastly, we conducted our third planned comparison between the enhanced and prevented conditions, we once again found a significant main effect of time, no main effect of condition, and a significant interaction effect. In line with our hypotheses, our results show that the level of mental imagery impacts the perceived risk of climate change—the more mental imagery used, the more the perceived risk of climate change was increased.

### Affective experience

#### Valence

Concerning affective experience, we expected that enhancing mental imagery would lead to a decrease in the level of positive affective valence of larger magnitude and a increase in the level of arousal of a larger magnitude, compared to both the spontaneous and prevented use of mental imagery (3). Subsequently, we also expected that preventing mental imagery would lead to different magnitudes of change in positive affective valence and arousal, when compared to the control and enhanced mental imagery condition (4). To answer these hypotheses, we again used split-plot ANOVAs (separate for emotional valence and arousal), with planned comparisons. To see if the magnitude of the change in the variables were different depending on the condition, our main interest was again the interaction effects between condition and time for each affective measure.

When comparing affective valence in all three conditions the results revealed a main effect of time, a main effect of condition, and an interaction effect. Thus, the results show that the participants level of positive valence decreased, however, as expected to different degrees in the different conditions. When comparing the conditions on reported valence post-manipulation with a simple main effect analysis (Sidak), no difference was found between the control and enhanced condition (*p* = 0.18), while the prevented condition differed significantly from both the enhanced (p < 0.001) and the control (p < 0.001) conditions.

When comparing valence in the spontaneous mental imagery condition (control) to the enhanced condition we found a main effect of time, no main effect of condition, but an interaction effect. The results indicate that although both conditions decreased in positive affective valence, this decrease was larger in the enhanced mental imagery condition.

Similarly, when comparing the spontaneous mental imagery condition (control) to the prevented condition we found a main effect of time, a main effect of condition, and an interaction effect. The results show that although positive valence decreased in both conditions, it decreased less in the prevented imagery condition.

Lastly, when comparing the enhanced and prevented conditions, we found a main effect of time, a main effect of condition, and an interaction effect. In line with our hypotheses, our results show that level of mental imagery used to process risky environmental events impacts the affective valence—the more mental imagery the participants used to process the events the more they decreased their positive affective valence.

#### Arousal

When comparing affective arousal in all three conditions the results revealed a main effect of time, no main effect of condition, however an interaction effect. Thus, the results indicate that the participants change in arousal level are dependent on the condition. When comparing the conditions on the reported arousal post-manipulation with a simple main effect analysis (Sidak), no difference was found between the control and enhanced condition (*p* = 0.146), while the prevented condition differed significantly from the enhanced (p = 0.042) and the control (p < 0.001) conditions.

When comparing the level of arousal in the spontaneous mental imagery condition (control) to the enhanced condition we found no main effect of time, no main effect of condition, and no interaction effect. The results indicate that there was no significant change in arousal in either the spontaneous mental imagery condition (control) or the enhanced condition. However, when comparing the spontaneous mental imagery condition (control) to the prevented condition we did find a main effect of time, and a main effect of condition, and an interaction effect. The results indicate that only the prevented mental imagery condition increased in level of arousal. Further supporting that only prevented condition changed the level of arousal, we found a main effect of time, no main effect of condition, but an interaction effect, when comparing the enhanced and prevented conditions. The results from the analyses show that only the prevented condition changed the affective arousal from pre-manipulation to post-manipulation, indicating that the mental rotation task, and not the mental imagery level led to the change in arousal level. In the pre-registration regarding hypothesis 4, we wrote that we did not specify a direction in affective arousal “direction not specified”. We did this because we were not sure if the task would be activating or de-activating. However, this was in some contradiction to hypothesis 3; since hypothesis 3 precedes hypothesis 4, we decided to remove this from the manuscript. This did not change the interpretation of the results since affective arousal did not seem to be changed by the level of mental imagery.

### Pro-environmental intention and impact belief

In addition to the main research question, we posed an additional question: Is there a relationship between perceived risk and behavioral intention and/or impact belief? Two additional exploratory research questions were posed in the pre-registration (see OSF), for analyses to answer these see the supplementary material. When comparing the level of pro-environmental intention post-manipulation using a one-way ANOVA, no difference was found between conditions (*p* = 0.41). Similarly, when comparing the level of impact belief post-manipulation using a one-way ANOVA, no difference was found between conditions (*p* = 0.87). Thus, these results suggest that changing the level of mental imagery and subsequent affective valence and perceived risk of climate change did not alter the participants' intention to act pro-environmental, nor impact their belief in how effective pro-environmental behaviors are in reducing the impact of climate change.

## Discussion

The literature on affect-as-information^2^, the affect heuristic^35^ and risk-as-feelings^3^ emphasize that affective experiences impact our risk perception. In this study, we examined the role of mental imagery in amplifying affective responses and its subsequent impact on the perceived risk of climate change. We systematically varied the level of mental imagery by either allowing for spontaneous mental imagery, enhancing it, or preventing it. As predicted, we found a causal link between the level of mental imagery, affect, and perceived risk of climate change. Our results provide novel evidence of the role that mental imagery plays in changing affect used as information when assessing climate change related risks.

Our results suggest that on average positive affect decreased and risk perception of climate change increased when processing the environmental risk events. However, in line with our hypotheses, we specifically find that an increased level of mental imagery was related to a larger decrease in positive affective valence and a higher increase in perceived risk of climate change. These results are in line with previous research showing a link between mental imagery, affect, and risk perception outside the domain of climate change^10,15,17^, and the results within the same domain but applying different methodologies^13,36^. However, as our approach allowed us to prevent visuospatial mental imagery processing, our results strengthened the evidence for causality. Concerning arousal, only the prevented mental imagery seemed to increase the arousal level, possibly reflecting the cognitive effort needed in the mental rotation task, rather than being associated with mental imagery.

We find no evidence that the mental imagery manipulation changed the intention to act pro-environmentally or the perceived impact of these behaviors. These results are in contrast with a study that identified a link between mental imagery, perceived risk of climate change, and pro-environmental intentions and behavior^24^. The failure to find these relationships, particularly regarding pro-environmental intention, could potentially be explained by the fact that we only measured intention post-manipulation which did not allow a comparison of degree of change between the conditions. However, the difference between our results and the previous study may also have to do with the difference in the mental imagery manipulations used. In Lee et al.^24^, participants read about the negative impact of climate change in their country and listed risk events that would happen in the future, potentially making the risk more psychologically close. The increased psychological proximity of the environmental risk events could be responsible for the increase in pro-environmental intention, although psychological proximity does not always lead to the expected effects^37^. In support of the impact of psychological proximity, earlier research has shown that environmental risk events presented (e.g., ice melting), that are perceived as psychologically distant, are indeed related to negative affect and higher perceived risk, but without leading to behavioral changes^38^. Lastly, the lack of an effect on pro-environmental intention and impact belief might be due to that perceived behavioral control (e.g., theory of planned behavior^39^) does not necessarily increase with increased perceived risk.

Our null finding suggests that there may be limitations to interventions aiming to increase pro-environmental behavior by increasing the perceived risk. However, previous research suggests that focusing on the consequences, particularly the health consequences of climate change, could help motivate pro-environmental behavior and policy support^40^. An alternative approach to strengthen the relationship between mental imagery, affect and pro-environmental behavior is focusing on more constructive mental imagery—research has shown that the ability to imagine the world post-sustainable changes to society is linked to environmental activism and adaptation behaviors^41,42^. More constructive mental imagery, could possibly also lead to a higher level of perceived behavioral control, and thus increasing the chance of pro-environmental behavioral changes.

Although the methodology in the current study allowed us to look at mental imagery processing at different levels, there are some limitations. One such limitation is that the conditions were not time matched. Future studies should control for this to make sure that the effects on valence and perceived risk are not due to the amount of processing time. However, our results indicate that the effect of the manipulations is not a result of the amount of processing time, since the control condition (shortest duration) had higher perceived risk and negative affective valence compared to the prevented condition (middle duration), which would not be the case if processing time drove the interaction effect. Also, previous research on mood and affect shows that most induced affect declines after a few minutes (e.g., 4–7), rendering it unlikely that the more time spent in the enhanced mental imagery condition to explain our main findings^43,44^.

Our control condition in which we allow for spontaneous imagery processing has benefits in that it increases the ecological validity of the results. However, there are potential confounding factors in the control condition such as the lower effort needed in this condition. However, we do not believe lower effort in the control condition could explain our results. Using mental rotation as visual-spatial interference allowed us to experimentally switch on and off mental imagery processing of environmental risk events^19,27,28^, however, the results indicate that this condition needed more cognitive effort than the mental imagery condition. Further, one could argue that the visuospatial interference served as a form of distraction from the risks. Future studies should measure affective experience and perceived risk when more time has passed, instead of only directly after the manipulation, to examine if the effects disappear when all individuals have had more time to process the risks.

Lastly, one could argue that mental imagery is difficult to separate from general affect associated with the risk, thus suggesting that our results only further support that affect is used as information in risk judgments. However, our findings show that the mental imagery manipulation was successful in inducing differentially vivid mental images related to the same environmental risks. Hence, like others, we argue that mental imagery can amplify affect^8,26^, as well as increase the perceived risk.

To conclude, our findings strengthen the support for a causal relationship between mental imagery, affective experience, and risk perception, specifically here in the context of climate change. We argue that mental imagery increases the affect associated with the risk event by creating a perceptual experience that mimics seeing the environmental risk events, and then accordingly generating affective responses that can serve as information when making affect-laden risk judgments. However, we failed to find a relationship between mental imagery and pro-environmental intention and impact beliefs. Our findings increase our understanding of how affect impacts risk perception, but future studies should investigate the possible implications of how changing risk perception through affective manipulations might impact environmental values and behavior.

## Supplementary Information

Supplementary Information.

## Data Availability

The datasets generated during and/or analyzed during the current study are available in the Open Science Framework, https://osf.io/k6vp9/?view_only=5aa191c0ad0041b8aed85df0ae9f25f0 [View only link]. The study was preregistered; the registration can be found at: https://osf.io/e26yf/?view_only=02b76bd281214815be970959799e1d18 [View only link].
