# Supplementary material for: A causal link between mental imagery and affect-laden perception of climate change related risks
Source: Sci Rep. 2023 Jun 21;13:10081. doi: 10.1038/s41598-023-37195-w (PMC10284903; doi:10.1038/s41598-023-37195-w)
Supplement: Supplementary file 1 — Supplementary Information. [file 41598_2023_37195_MOESM1_ESM.docx]

Supplementary –

A causal link between mental imagery and affect-laden perception of climate change related risks

Hulda Karlsson ^a*^, Erkin Asutay ^b^, Daniel Västfjäll ^c, d^

^a^ Department of behavioural sciences and learning, JEDI-lab, Linköping University, Linköping, Sweden. ORCID: 0000-0001-6890-3298

*Corresponding author. Linköping University, Department of behavioural sciences and learning, House I:3, Campus Valla, SE 581 83 Linköping, Sweden.

Email: hulda.karlsson@liu.se

^b^ Department of behavioural sciences and learning, JEDI-lab, Linköping University, Linköping, Sweden. ORCID: 0000-0002-4257-6732

^c, d^ Department of behavioural sciences and learning, JEDI-lab, Linköping University, Linköping, Sweden and Decision Research Eugene, Oregon, USA. ORCID: 0000-0003-2873-4500

Contests

[1. Manipulation check 3](#_Toc137467076)

[2. Order effects 4](#_Toc137467077)

[3. Vividness 5](#_Toc137467078)

[4. Non-parametric tests 6](#_Toc137467079)

[5. Risk recipient 8](#_Toc137467080)

[6. Correlation dependent measures 9](#_Toc137467081)

[7. Hierarchical linear regression inter-manipulation ratings 11](#_Toc137467082)

# Manipulation check

Post-manipulation all participants answered three self-report measure items “To what degree did you do the following when you read the environmental events statements?”, on a scale between 0 (To a low degree) and 100 (To a high degree). The three items were: (1) Read the words in the statement; (2) Engage in mental rotation; (3) Mentally imagine the environmental events.

When comparing the manipulation check between the three conditions participants reported engaging the most in mental imagery during in the enhanced condition, followed by the instinctive and lastly the prevent mental imagery condition. This is the main manipulation check sense the study is concerned with level of mental imagery. However, regarding mental rotation both active conditions differed from the instinctive condition, however not from each other. Lastly, only the prevented mental imagery condition differed from the instinctive condition concerning to what degree they read the words in each environmental event statement.

**S1** *Manipulation check split per condition*

| Manipulation check | Spontaneous mental imagery (control) | Enhanced mental imagery | Prevented mental imagery |
| --- | --- | --- | --- |
| Read the words | 93.88^a^  (12.02) | 93.37^a^  (11.85) | 90.92^b^  (15.96) |
| Mental rotation | 71.96^a^  (28.19) | 82.10^ab^  (23.88) | 83.73^a^  (26.56) |
| Mentally imagine | 85.88^a^  (19.04) | 91.43^b^  (15.04) | 75.63^c^  (26.04) |

**Note.** The degree participants engaged in the above behaviors during the manipulation split by condition. The values in brackets are the standard deviation. The table all participants that were used in the main analysis (*N* = 1000). Different subscript letter indicates significant difference (at least 0.05 level) in change scores as determined by Tukey post-hoc.

# Order effects

No main effects or interactions were found for the order of how the environmental risk events were presented for any of the dependent variables: perceived risk (main effect, *p* = .46; interaction, *p* = .55), or affective experience, valence (main effect, *p* = .79; interaction, *p* = .79), arousal (main effect, *p* = .56; interaction, *p* = .74).

# Vividness

We compared the level of vividness between conditions for each environmental risk event individually, using a one-way ANOVA. The conditions varied significantly on the level of vividness on all risk events. Tukey post-hoc analysis reveal that all conditions varied significantly in the expected direction, where participants in the prevented condition had the least vivid images, followed by the instinctive imagery condition (control) and with the enhanced imagery condition creating the most vivid images.

**S2** *Inter-manipulation vividness ratings split per condition and environmental risk event*

| Environmental risk event | Spontaneous mental imagery (control) | Enhanced mental imagery | Prevented mental imagery |
| --- | --- | --- | --- |
| Water pollution increasing | 2.99  (1.23) | 3.82  (0.91) | 2.40  (1.24) |
| Polar ice caps melting | 3.29  (1.22) | 3.68  (0.96) | 2.63  (1.21) |
| Wildfires increasing | 3.65  (1.12) | 4.02  (0.85) | 2.78  (1.28) |
| Floods increasing | 3.35  (1.20) | 3.89  (0.86) | 2.50  (1.24) |

**Note.** Vividness of mental imagery divided by environmental risk event and condition. The values in brackets are the standard deviation. The table all participants that were used in the main analysis (*N* = 1000). All three conditions differed significantly on all environmental risk events (0.001 level) as determined by Tukey post-hoc.

# Non-parametric tests

**Within**

***Perceived risk***

Pre-manipulation ratings were compared to post-manipulation ratings in the instinctive mental imagery condition (control). On average participants increased in their perceived risk, z = -8.74, *p* < .001. Pre-manipulation ratings were compared to post-manipulation ratings in the enhanced mental imagery condition. On average participants increased in their perceived risk, z = - 10.90, *p* < .001. Pre-manipulation ratings were compared to post-manipulation ratings in the prevented mental imagery condition. On average participants increased in their perceived risk, z = - 5.83, *p* < .001.

***Valence***

Pre-manipulation ratings were compared to post-manipulation ratings in the instinctive mental imagery condition (control). On average participants increased in their valence, z = - 13.75, *p* < .001. Pre-manipulation ratings were compared to post-manipulation ratings in the enhanced mental imagery condition. On average participants increased in their valence, z = - 13.56, *p* < .001. Pre-manipulation ratings were compared to post-manipulation ratings in the prevented mental imagery condition. On average participants increased in their valence, z = - 8.44, *p* < .001.

***Arousal***

Neither participant in the instinctive menta imagery condition (*p* = .19) or the enhanced mental imagery condition (*p* = .13) changed in their arousal level from pre-manipulation to post-manipulation. Pre-manipulation ratings were compared to post-manipulation ratings in the prevented mental imagery condition. On average participants increased in their arousal, z = - 6.34, *p* < .001.

**Between**

***Perceived risk***

The instinctive imagery condition did not differ from the enhanced mental imagery condition (*p* = .23) or the prevented mental imagery condition (*p* = .86) pre-manipulation. The active conditions did not differ from each other pre-manipulation (*p* = .20).

The instinctive imagery condition did not differ from the enhanced mental imagery condition (*p* = .76) or the prevented mental imagery condition (*p* = .50) post-manipulation. The active conditions did not differ from each other post-manipulation (*p* = .34).

***Valence***

The instinctive imagery condition did not differ from the enhanced mental imagery condition (*p* = .14) or the prevented mental imagery condition (*p* = .73) pre-manipulation. The active conditions did not differ from each other pre-manipulation (*p* = .23).

The instinctive imagery condition did not differ from the enhanced mental imagery condition (*p* = .07), however it differed from the prevented mental imagery condition, z = - 6.25, *p* <.001 post-manipulation. The active conditions also differ significantly from each other post-manipulation, z = - 7.85, <.001.

***Arousal***

The instinctive imagery condition did not differ from the enhanced mental imagery condition (*p* = .72) or the prevented mental imagery condition (*p* = .70) pre-manipulation. The active conditions did not differ from each other pre-manipulation (*p* = .95).

The instinctive imagery condition did not differ from the enhanced mental imagery condition (*p* = .20), however it differed from the prevented mental imagery condition, z = - 3.86, *p* <.001 post-manipulation. The active conditions also differ significantly from each other post-manipulation, z = - 2.39, *p* =.02

# Risk recipient

When completing the same analyses as in the main analyses, but split by risk recipient (self, others, non-human), we found a mostly similar pattern of results for risk for self and non-human.

In contrast the pattern of perceived risk for others differed from the aggregated measure, the perceived risk decreased when mental imagery was prevented. No significant change was detected in the other two conditions. Thus, these exploratory results suggest that how we perceive the risk of climate change for others differ from how we perceive it for ourselves and non-human species.

| Recipient | Main effect of time | Main effect of condition | Interaction effect |
| --- | --- | --- | --- |
| Self all | *** |  | *** |
| *Control vs Enhanced* | *** |  | ** |
| *Control vs Prevented* | *** |  | ** |
| *Enhanced vs Prevented* | *** |  | *** |
| Other all | * |  | *** |
| *Control vs Enhanced* |  |  | * |
| *Control vs Prevented* | *** |  | ** |
| *Enhanced vs Prevented* |  |  | *** |
| Non-human all | *** |  | *** |
| *Control vs Enhanced* | *** |  | * |
| *Control vs Prevented* | *** |  |  |
| *Enhanced vs Prevented* | *** |  | *** |

**S3** *Two-way ANOVA with contrast split per risk recipient and condition*

**Note.** Results from two-way ANOVAs split per risk recipient and condition. *** = significance level of <.001, ** = significance level of <.01, * = significance level of .05. Empty field = non-significant effect.

**S4** *Mean perceived risk split by recipient and time*

| Recipient | Instinctive mental imagery (control) | | Enhanced mental imagery | | Prevented mental imagery | |
| --- | --- | --- | --- | --- | --- | --- |
|  | 1 | 2 | 1 | 2 | 1 | 2 |
| Self | 65.43 | 68.87 | 62.86 | 68.68 | 65.28 | 67.02 |
| Other | 73.30 | 72.56 | 70.84 | 72.30 | 73.45 | 70.67 |
| Non-human | 79.32 | 81.38 | 78.29 | 82.04 | 78.98 | 80.13 |

**Note.** This includes all participants used in the analyses (*N* = 1000). 1 = Pre-manipulation; 2 = Post-manipulation.

# Correlation dependent measures

Perceived risk and affective valence had a significant correlation in all conditions; however, the strength of the correlation was significantly different in the enhanced mental imagery condition compared to the other two conditions. This indicates that valence was more related to the risk perception of climate change in the enhanced condition.

Perceived risk and arousal were only significantly correlated in the control condition and the strength of the correlations did not differ between any of the conditions.

Perceived risk and vividness correlated significantly in all conditions. However, the enhanced and control conditions differed in the strength of the correlation. This indicate that the vividness of mental imagery was more related to perceived risk in the control than the enhanced condition. This could be explained by the reduced higher level of vividness in the enhanced condition.

Perceived risk and pro-environmental intention correlated significantly in all conditions. Further, there was no difference in the strength of the correlation.

Perceived risk and impact belief correlated significantly in all conditions. Further, there was no difference in the strength of the correlation.

**S5** Spearman *correlations between post-manipulation perceived risk, affective valence, arousal, pro-environmental intention, impact belief and inter-manipulation vividness of mental imagery split per condition*

|  | Spontaneous mental imagery (control)  (*N* = 346) | | | | | Enhanced mental imagery  (*N* = 308) | | | | | Prevented mental imagery  (*N* = 346) | | | | |
| --- | --- | --- | --- | --- | --- | --- | --- | --- | --- | --- | --- | --- | --- | --- | --- |
| Variable | 1 | 2 | 3 | 4 | 5 | 1 | 2 | 3 | 4 | 5 | 1 | 2 | 3 | 4 | 5 |
| 1. Risk | - |  |  |  |  | - |  |  |  |  | - |  |  |  |  |
| 2. Valence | -.254**^a^ | - |  |  |  | -.407**^b^ | - |  |  |  | -.168**^a^ | - |  |  |  |
| 3. Arousal | .165**^a^ | .281**^a^ | - |  |  | .078^a^ | .073^b^ | - |  |  | .050^a^ | .483**^c^ | - |  |  |
| 4. Vividness | .288**^a^ | -.116*^a^ | .287**^a^ | - |  | .159**^b^ | .009^ab^ | .301**^a^ | - |  | .178**^ab^ | .153**^c^ | .174**^a^ | - |  |
| 5. Intention | .486**^a^ | -.228** | .153** | .340** | - | .497**^a^ | -.235** | .174** | .098 | - | .421**^a^ | <.001 | .084 | .215** | - |
| 6. Belief | .446**^a^ | -.067 | .221** | .313** | .502** | .518**^a^ | -.193** | .146* | .155** | .560** | .432**^a^ | .114* | .180** | .222** | .557** |

**Note.** The vividness measure consists of the inter-manipulation ratings averaged. The analyses includes all participants used in the analyses (*N* = 1000). Different superscript letters indicated significant differences (0.05 level) in strength of the correlation. Significant correlation coefficient indicated by: ** = .001, * = .05.

# Hierarchical linear regression inter-manipulation ratings

We conducted a hierarchical liner regression model with the perceived risk post-manipulation as the dependent variable. In model 1, we only included the pre-manipulation perceived risk-ratings to control for the baseline level of perceived risk (model 1 was significant *p* <.001). In the second model, we included the inter-manipulation ratings of affective valence, arousal, and vividness of mental imagery, averaged over all four environmental risk events. In the second model we also included the dummy variable comparing against the enhanced mental imagery condition. Lastly, the second model also included the interaction between the dummy coded condition variables and the inter-manipulation ratings. Model 2 was also significant (*p* <.001) however the included variables seem to have added little predictive value, where model 1 (only perceived risk pre-manipulation) explained as much as 92.8 % (adjusted *R*) of the variance in the perceived risk post-manipulation, the second model only added an additional 4% (*R*^2^ change).

Looking at the individual predictors in model 2 (see table 5), inter-manipulation ratings of valence and arousal predicted perceived risk post-manipulation. The inter-manipulation vividness rating was not significant. Further, neither of the dummy variables were significant predictors, indicating that the condition did not explain the difference post-manipulation, this was however not completely unexpected since we did not find significant differences between conditions post-manipulation in the main analyses. No interaction was a significant predictor indicating that the difference between conditions in inter-manipulation ratings did not explain post-manipulation risk perception.

**S6** *Model 2* *hierarchical linear regression on perceived risk of climate change post-manipulation controlling for pre-manipulation ratings of perceived risk, with inter-manipulation ratings of affective valence, arousal, and vividness as predictors*

| **Variabel** | ***B*** | **β** | ***t*** | ***p*** |
| --- | --- | --- | --- | --- |
| Valence | - 0.50 | -.05 | -2.89 | .004 |
| Arousal | 0.38 | .03 | 2.10 | .036 |
| Vividness | 0.79 | .03 | 1.45 | .147 |
| Control dummy | 1.44 | .03 | 0.52 | .605 |
| Valence * Control dummy | 0.02 | .02 | 0.74 | .462 |
| Arousal * Control dummy | 0.01 | -.01 | -0.23 | .819 |
| Vividness * Control dummy | -0.01 | -.06 | -1.38 | .169 |
| Prevented dummy | -2.06 | .04 | -0.76 | .439 |
| Valence * Prevented dummy | -0.03 | .06 | 1.89 | .059 |
| Arousal * Prevented dummy | -0.03 | -.02 | -0.67 | .504 |
| Vividness * Prevented dummy | -0.03 | -.02 | -0.68 | .491 |

**Note.** Coefficients from hierarchical multiple regression model 2. Hierarchical regression predicting the dependent variable perceived risk of climate change. The inter-manipulation ratings of valence, arousal and vividness were averaged. All participants were included in the analyses (*N* = 1000).
